# Removal of Ampicillin by Heterogeneous Photocatalysis: Combined Experimental and DFT Study

**DOI:** 10.3390/nano11081992

**Published:** 2021-08-03

**Authors:** Lenka Belhacova, Hana Bibova, Tereza Marikova, Martin Kuchar, Radek Zouzelka, Jiri Rathousky

**Affiliations:** 1 Department of Electrochemical Materials, J. Heyrovsky Institute of Physical Chemistry of the CAS, Dolejskova 3, 18223 Prague, Czech Republic; hana.bibova@jh-inst.cas.cz; 2 Center for Innovations in the field of Nanomaterials and Nanotechnologies, J. Heyrovsky Institute of Physical Chemistry of the CAS, Dolejskova 3, 18223 Prague, Czech Republic; tereza.marikova@jh-inst.cas.cz (T.M.); radek.zouzelka@jh-inst.cas.cz (R.Z.); 3 Forensic Laboratory of Biologically Active Substances, Department of Chemistry of Natural Compounds, University of Chemistry and Technology Prague, Technicka 3, 16628 Prague, Czech Republic; kuchara@vscht.cz

**Keywords:** water treatment, photocatalysis, TiO_2_, DFT, ampicillin, antibiotics

## Abstract

A long-term exposition of antibiotics represents a serious problem for the environment, especially for human health. Heterogeneous photocatalysis opens a green way for their removal. Here, we correlated the structural-textural properties of TiO_2_ photocatalysts with their photocatalytic performance in ampicillin abatement. The tested nanoparticles included anatase and rutile and their defined mixtures. The nominal size range varied from 5 to 800 nm, Aeroxide P25 serving as an industrial benchmark reference. The degradation mechanism of photocatalytic ampicillin abatement was studied by employing both experimental (UPLC/MS/MS, hydroxyl radical scavenger) and theoretical (quantum calculations) approaches. Photocatalytic activity increased with the increasing particle size, generally, anatase being more active than rutile. Interestingly, in the dark, the ampicillin concentration decreased as well, especially in the presence of very small nanoparticles. Even if the photolysis of ampicillin was negligible, a very high degree of mineralization of antibiotic was achieved photocatalytically using the smallest nanoparticles of both allotropes and their mixtures. Furthermore, for anatase samples, the reaction rate constant increases with increasing crystallite size, while the degree of mineralization decreases. Importantly, the suggested degradation pathway mechanism determined by DFT modeling was in very good agreement with experimentally detected reaction products.

## 1. Introduction

**Figure 1:**
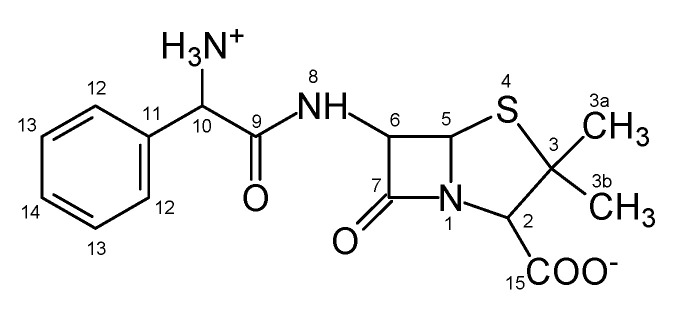
Ampicillin molecule in the zwitterionic form.

Due to their long-term toxic effects, antibiotics are considered a global environmental problem [1,2,3,4,5]. Ampicillin (Figure 1), a representant of widely used pharmaceuticals of the penicillin group, is discharged to the environment in two main ways: (i) through the human and animal bodies; although ampicillin is partially metabolized by hydrolysis of the beta-lactam ring to penicilloic acid, its majority is excreted unchanged and enters sewage effluents (municipal wastewaters), and, (ii) through the agrochemical industry as important sources of water and soil contamination.

Since ampicillin is hardly biodegradable, conventional purification methods used in wastewater treatment plants (WWTPs) were found to be inefficient to achieve its complete degradation, and its residues still remained in secondary treated wastewater effluents [6,7,8]. These potentially harmful compounds are subsequently discharged into the environment, where they can affect aquatic and terrestrial ecosystems.

Regarding the above-mentioned facts, implementation of non-biological methods in WWTPs to achieve the complete removal of biologically active water pollutants becomes an increasingly urgent requirement. Economically viable and environmentally acceptable solutions that can be implemented to existing practices are preferred. Advanced oxidation processes (AOPs), based on reactions generating reactive species, e.g., hydroxyl radicals that oxidize organic molecules up to their complete mineralization, have proved to be very efficient in the degradation of many organic pollutants in diverse wastewaters, including antibiotics [9,10] among other pharmaceuticals [11,12,13,14,15,16].

Concerning the removal and degradation of beta-lactam antibiotics, procedures combining H_2_O_2_ and UV irradiation treatment, radiolysis [17], ozonolysis [18], Fenton- and photo-Fenton methods [8,19], semiconductor photocatalysis [20,21,22,23], or their combination [24] were studied. Many of them led to the ampicillin disposal from aqueous solutions, but they were often associated with practical disadvantages (depletion of the radical source, need for UVC radiation, high material costs, etc.). In this respect, heterogeneous photocatalysis, especially in a set-up with photocatalyst deposited on a modifiable support, appears to be a promising method that can be used in continuous operation in small water treatment plants.

However, to obtain a meaningful assessment of the applicability of the heterogeneous photocatalysis, all the main crucial aspects of this technology should be addressed at once, which is a substantial novelty of our study compared to other relevant published studies. Therefore, our study has aimed at this complex task, having dealt with

- Determining the correlations between the properties of TiO_2_ photocatalysts (particle size, degree of agglomeration, crystallinity, phase composition) and their photocatalytic performance, based on the use of extensive sample bank,
- Not only the photocatalysis itself but also with accompanying processes, namely sorption on the photocatalytic surface and photolysis,
- The coupling of the experimental research and DFT calculations to obtain a deep insight into the mechanism of the whole process, which is of major importance for the assessment of the effect of other components present simultaneously in the water,
- The achievement of practically complete mineralization to minimize the risk of potentially toxic organic products.

Of special importance was the identification of products by high-performance UPLC/MS/MS analysis, which were suggested by the DFT calculations. The non-specific attack of reactive species generated by the photocatalytic process on complex molecules renders any prediction very difficult. Therefore, the agreement achieved is especially valuable and substantially helps to select the most real pathway of antibiotic degradation.

## 2. Materials and Methods

### 2.1. Characterization of Photocatalytic Powders

All TiO_2_ nanopowders were purchased from US Research Nanomaterials (≥99.5% purity). The samples included both pristine allotropic forms of TiO_2_ (rutile and anatase) and their defined mixtures. According to the manufacturer, the particle size varied from 5 to 800 nm. Aeroxide *P25* was purchased from Evonik (≥99.5% purity). In the labeling of the samples, the letters *A*, *R*, *A+R,* and *AM* designate anatase, rutile, anatase+rutile mixed phases, and “amorphous” TiO_2_ as well as the number indicating the nominal particle size, as given by the producer.

The structural and textural properties of the nanoparticles were determined by a combination of several techniques. Surface morphology was followed with scanning electron microscopy (SEM, JEOL JSM 5500LV) combined with high-resolution transmission electron microscopy (HR-TEM, JEOL JEM-2100Plus). Crystallinity (structure and phase composition) of the titania dioxide samples was evaluated by powder X-ray diffraction (pXRD) using PANalytical X′Pert PRO diffractometer equipped with Co anode in the Bragg-Brentano geometry and multichannel X′Celerator detector. The surface area of powders was determined from nitrogen sorption isotherms at the boiling point of liquid nitrogen using a Micromeritics 3Flex apparatus. Prior to the measurements, the samples were degassed at 250 °C overnight.

### 2.2. Measurement of Degradation Kinetics, Sorption, and Photolysis

The experiments were carried out in a photoreactor set-up of own design and construction, capable of performing series of experiments under identical conditions, reducing thus random effects. It is equipped with 10 fluorescent black lamps (λ_max_ = 365 nm, 8 W, Sylvania), the flux density at the probe level being 6.24 mW·cm^−2^ measured by UVA probe (ILT 1400-A Photometer, probe UVA #28949). Irradiated suspensions in beakers (50 mL) were stirred by magnetic stirrers (450 rpm), and 0.5 mL aliquots were taken for the HPLC analysis.

The concentration of ampicillin (AMP, anhydrous, Sigma-Aldrich, Prague, Czech Republic) was determined by HPLC (Agilent Technology 1200 Series; column Kinetex Polar C18 2.6 μm; isocratic elution: 75% H_2_O with 0.1% H_3_PO_4_, (A) 25% methanol (B), flow rate 1 mL/min, injection volume 100 µL, detection wavelength 224 nm), using a gradient method (0–2 min: 90% A; 2–12 min: 65% A; 12–16 min: 35% A). The solvents (Fluka Chemical) were of the HPCL gradient grade. Prior to each analysis, the samples were centrifuged (14,500 rpm, 10 min). TOC analysis was performed with a Shimadzu TOC-L Analyzer, using 20 mL of centrifuged solution. The obtained kinetic curves were analyzed using the 1st order reaction model, the rate constants being calculated by a non-linear curve fitting using a Levenberg–Marquardt algorithm.

As the TiO_2_ nanopowders were not degraded due to the photocatalytic process, they were suitable for re-use. However, their quantitative separation required the use of high-performance ultracentrifuge. In our case, we used an Optima XPN-100 ultracentrifuge (Beckman Coulter), equipped with a titanium rotor Ti-70, the separation conditions: 70,000 rpm, 15 min, temperature 15 °C. Finally, fresh pollutant solution was added to the separated photocatalytic nanoparticles, and the re-use test was performed, the resulted reaction rate constant differing from the original one by 8%.

### 2.3. Experimental Detection of Photocatalytic Products by UPLC/MS/MS

The degradation products analysis was performed using an Agilent Technologies UPLC/MS/MS system consisting of a 6550 iFunnel Q-TOF detector and an Infinity 1290 UPLC system. Each sample taken was analyzed twice—immediately after collection and over a longer period of time (4–6 h after the first MS/MS analysis).

The photodegradation products separation was performed on an Eclipse plus C18 LC column (1.8 μm; 100 × 2.1 mm; Agilent Technologies) heated to 45 °C. The mobile phase consisted of 0.1% formic acid and 5 mM ammonium formate in water (A) and methanol with 0.1% formic acid (B), and the gradient elution was performed as follows (all steps linear): 0 min—95:5 (A:B); 7 min—50:50; 9 min—0:100; 12 min—0:100; 12.2 min—95:5; 13 min—95:5. The flow rate of the mobile phase was 0.3 mL·min^−1^ and the sample injection was 2 μL.

The samples were ionized by electrospray ionization in positive mode. The *m*/*z* values in the range of 50–1050 were monitored in MS/MS mode. Agilent MassHunter Qualitative Analysis software was used to evaluate the obtained data. To evaluate the MS analysis, adducts of the photodegradation molecules with H^+^, Na^+^, K^+^, CH_3_OH^+^, and NH4^+^ ions were set.

### 2.4. Quantum-Chemical Calculations

For geometry optimization, the DFT method at the B3LYP/6-31G(d) level in water solvent was used (default model IEFPCM), as implemented in commercially available software package Gaussian 09. This level of theoretical approach was chosen to characterize comparative trends and effects. All geometries were optimized until default convergence criteria were met, and for transition states, the frequency analysis was performed, followed by the examination of intrinsic reaction coordinate (irc) to ensure that the transition structures were located between the correct energy minima.

The activation energy of a reaction was calculated (at 298.15 K and 1 atm set as default) as a difference of calculated Gibbs free energies between a reactant and a transition state. The reaction Gibbs free energy was expressed as a difference between the Gibbs free energies of a product and a reactant. Negative values of ΔG represent exothermic reactions.

## 3. Results and Discussion

### 3.1. Characterization of Photocatalysts

Table 1 summarizes the structure and texture characteristics of all the TiO_2_ powders used. 

The phase content and the size of coherent regions were determined by the analysis of X-ray diffractograms, the broadening of the diffraction lines being interpreted only with regard to the crystal size, the microscopic deformations being neglected.

The data show that the pristine anatase and rutile samples contained exclusively the relevant phase. The content of these phases in mixtures, however, did not correspond to the data provided by the producer. Finally, the “amorphous” TiO_2_ powder was found to contain some proportion of the anatase phase.

Concerning the particle size, a comprehensive comparative study using three physically different experimental techniques was carried out, including the X-ray diffraction, nitrogen sorption, and HR-TEM microscopy. Surface areas calculated from sorption data using the BET method were converted to mean particle sizes using the spherical model approximation.

Comparison of the particle size determined by the techniques mentioned enabled to draw some interesting conclusions concerning the morphological properties of the powders. Let us start with the anatase series. The closeness of results obtained by all three methods shows that the well-separated particles of *A5* contain only one crystal, although its mean size was larger than the size provided by the producer. The same applies to *A15*, for which there was a reasonable agreement with the producer’s data. For samples containing larger particles, *A100* and *A800*, there was a substantial disagreement among the three methods, which indicates a more complex morphology of these materials. The coherent regions determined by the X-ray diffraction, as well as the particle size determined by N_2_ sorption and HRTEM, were much smaller than the nominal size provided by the producer. Clearly, these materials consisted of particles containing smaller crystals.

Concerning the rutile samples, *R30* contained crystals of ca 30 nm in size, which were aggregated to about three times larger particles as follows from the comparison of the XRD and sorption data. Samples *R100* and *R800* exhibited surprisingly similar particle sizes as determined from the sorption and HR-TEM measurements. Neither size, however, corresponded to the nominal one.

The particle size of samples containing both anatase and rutile *A+R2* and *A+R3* roughly corresponded to the nominal value of 20 nm with a relatively good agreement among the three experimental techniques used. The sample *A+R1* falls out of line as both the size of coherent regions and particle size determined by the sorption and HR-TEM were larger than the nominal 20 nm.

Finally, the “amorphous” sample *AM5*, even if not fully amorphous, showed particle size, which was close to the nominal value. This material consisted of well-separated particles, which contained only one coherent region, except for potentially small content of larger aggregates.

### 3.2. Sorption, Photolysis, and Photocatalysis of Ampicillin

To determine the true photocatalytic efficiency of the TiO_2_ samples, two additional phenomena should be considered, i.e., the removal of ampicillin by sorption on the surface of TiO_2_ particles and its degradation by direct photolysis.

#### 3.2.1. Sorption

**Figure 2:**
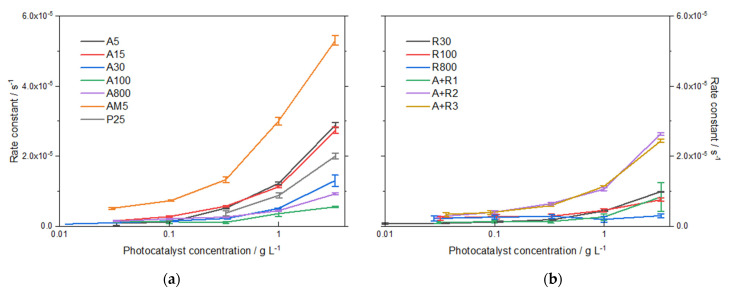
Rate constants of the ampicillin sorption in aqueous suspensions of all photocatalytic powders (**a**,**b**).

Under otherwise identical conditions, but without irradiation, ampicillin concentration in aqueous suspensions of TiO_2_ powders was found to decrease with time. The decrease of ampicillin concentration followed the 1^st^ order kinetics, and the sorption depended on the photocatalyst type, its crystallite size and concentration (Figure 2).

The data showed that with increasing photocatalyst concentration, the orption increased. Both allotropes of TiO_2_ showed higher sorption for smaller particle sizes. Similar trend was observed for *A+R2* and *A+R3* mixtures, and the anatase samples *A5* and *A15*, as corresponded to their similar particle size (Table 1). The highest sorption was observed for *AM5*, characterized by the smallest particles. 

HPLC/UV analysis of TiO_2_ suspensions with ampicillin in the course of time showed the formation of two degradation intermediates, similarly to Peterson et al. [25], who ascribed them to isomers and/or degradation products of penicilloic acid.

#### 3.2.2. Photolysis

Changes in the composition of ampicillin solution, irradiated in the absence of photocatalyst, were monitored by HPLC. No significant decrease in ampicillin concentration was observed after 5 h irradiation, consistently with the low extinction coefficient in its absorption spectrum. The corresponding rate constant of the photolytic decomposition was 4.29 ± 0.25 × 10^–6^ s^–1^.

#### 3.2.3. Photocatalysis

**Figure 3:**
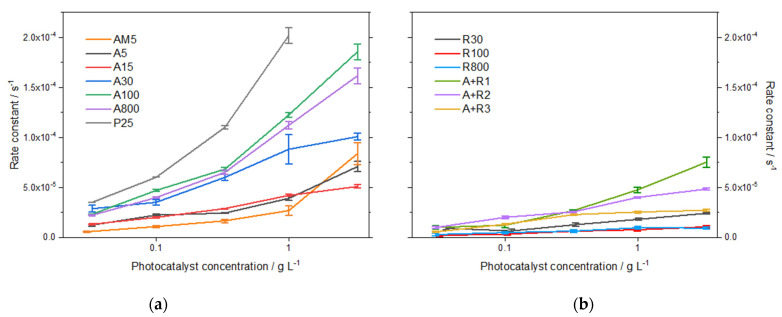
Rate constants of ampicillin photocatalytic degradation in aqueous suspensions of all photocatalytic powders (**a**,**b**).

Degradation of ampicillin in irradiated photocatalyst suspensions followed pseudo-first-order kinetics. The values of rate constants (Figure 3) were obtained by subtraction of the corresponding rate constants of sorption from the overall degradation process. The comparison of the two crystal modifications of the same particle size showed, as expected, higher activity of anatase powders compared to rutile. Amorphous TiO_2_ showed a relatively high total degradation activity, which can be partly attributed to strong sorption effects (about 50%). In the case of mixtures, the most active mixture was *A+R1*, composed mainly of anatase (Table 1, 55%) with a crystallite size larger than in the other two mixtures. The rate constant of ampicillin degradation in the suspension of *P25* (1.0 g L^–1^) was determined as 1.7 × 10^–4^ s^–1^. It can be concluded that with a larger crystallite size, the photocatalytic activity of the studied powders increased.

From our experiments, a clear-cut correlation follows. Figure S7 shows that the reaction rate constant increases with increasing anatase crystallite size. This variation can be expressed by the following linear equation:

Reaction rate × 10^4^/s^−1^ = 0.196 × 10^4^/s^−1^ + 0.015 × 10^4^/s^−1^ nm^−1^ * crystallite size/nm.

This equation is valid in the range of crystallite size from 5 to 80 nm.

A similar observation was reported by Zouzelka et al. [26], who described the effect based on the different nature of the two competing parallel primary photo-processes—charge carrier recombination and interfacial charge transfer, following second-order and pseudo-first-order kinetics, respectively. Assuming the formation of only one electron-hole pair at a time, their actual concentration decreases with larger particles. Since internal recombination is a second-order kinetic process, its rate is progressively reduced compared to interfacial charge transfer processes.

#### 3.2.4. Mineralization

Efficient mineralization of organic pollutants is a parameter of extreme importance in the environmental application of photocatalysis. Despite promising results of previous studies dealing with ampicillin degradation, some observations suggest that in the early stages of the treatment, toxicity towards bacteria increased [17,27]. The reason can be due to the increased hydrophilicity of formed intermediates, and, therefore, higher damage of bacterial cells occurred. Therefore, analysis of degradation products and determination of reaction mechanism appears necessary.

**Figure 4:**
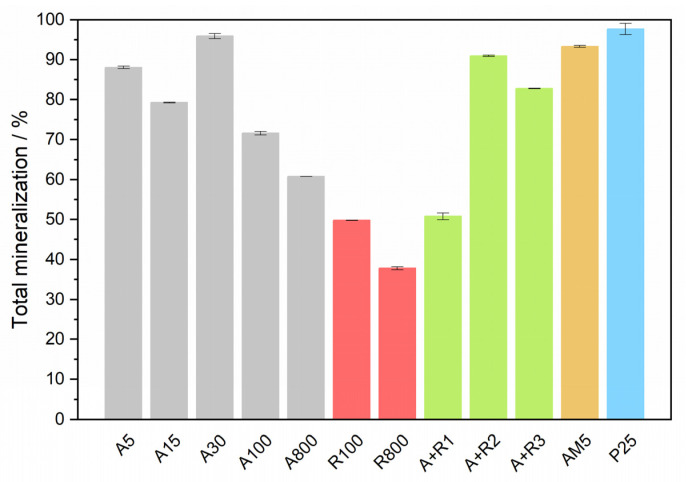
Degree of mineralization of ampicillin after long-term irradiation of 26 h.

Results indicate that nanoparticles of a similar crystallite size showed comparable activity in ampicillin mineralization. A high degree of mineralization after 5 h of 30–40% was observed for anatase *A5*, *A15*, and *A30*, and for its mixtures with rutile *A+R2* and *A+R3,* the highest value of 44% being obtained for *P25*. On the contrary, all rutile samples appeared much less efficient, similar to the anatase crystallites of larger size. The apparent discrepancy between the efficiency of ampicillin degradation and its total mineralization observed for anatase samples can be explained by the mechanism of ampicillin degradation. The chromatograms in Figure S1 show that the concentrations of intermediates in the irradiated ampicillin solutions in the presence of powders *A5* and *A100* significantly differ. The extent of adsorption on *A5* particles is higher than in the case of *A100*, therefore, it can be assumed that direct oxidation by h^+^ plays an important role. Since this process results, according to our theoretical calculation, in the immediate decarboxylation of the ampicillin molecule and the loss of one carbon atom, the reduction in TOC for the smaller anatase crystallite size would proceed more rapidly. In Figure 4, the degree of mineralization after long-term irradiation of 26 h is summarized for studied photocatalysts. The values of TOC correspond to the above observation, and for several photocatalysts, e.g., *A30*, *A+R2*, and *AM5*, almost complete mineralization was achieved.

From our experiments, clear-cut correlation follows. Figure S7 shows that mineralization decreases with increasing anatase size. This variation can be expressed by following linear equation:

Mineralization/% = 93/% − 0.26/% nm^−1^ * crystallite size/nm.

This equation is valid in the range of crystallite size from 5 to 80 nm.

### 3.3. Mechanism of Photocatalytic Degradation of Ampicillin

#### 3.3.1. Experimental Study

A comparative kinetic study was performed with *A100* and *R30* powders, characterized by low adsorption of ampicillin. Seven major peaks corresponding to the primary intermediates were identified (Figure S1). Their concentration has been estimated for comparative reasons, assuming that they have a similar structure and, therefore, spectral properties (extinction coefficient at the detection wavelength) as ampicillin. That is why it was possible to calculate the relative (and mutually comparable) rate constants of formation and degradation of each intermediate (Table S1). A comparison of the data shows that in the case of anatase, some products are formed at about twice the rate. The appearance of the same products was suppressed by adding isopropyl alcohol (hydroxyl radical scavenger) to the irradiated suspension (see the next paragraph), indicating that they are hydroxylated products of ampicillin. It can be concluded that in the case of anatase, the formation of hydroxylated products is preferred, while for rutile, other mechanisms are involved in the degradation, such as direct oxidation of the pollutant adsorbed on the surface of the particles [28,29].

#### 3.3.2. Photocatalytic Degradation with OH Radical Scavenger

**Figure 5:**
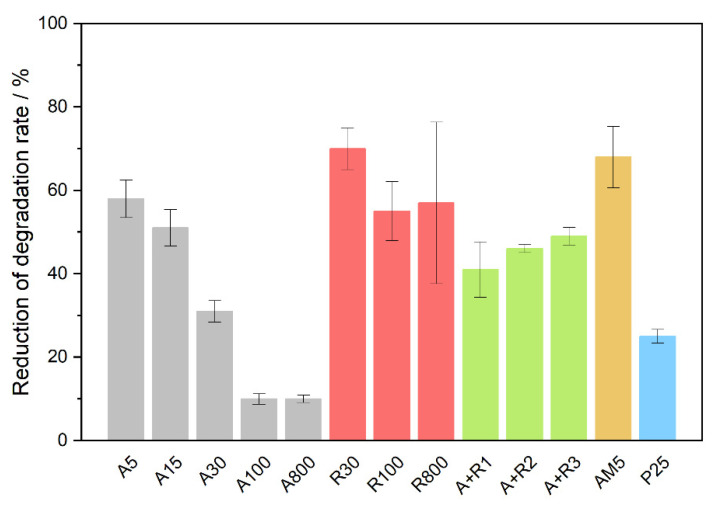
Reduction of rate constants of ampicillin degradation due to the isopropanol addition. The reduction was calculated as 100*(1 − k_IPA_/k).

As discussed in previous paragraphs, the extent of ampicillin adsorption on some of the studied materials is considerable and can play an important role in its degradation mechanism. Direct oxidation of pollutant molecules adsorbed on the particle surface thus proceed parallel to oxidation induced by hydroxyl radical attack. Therefore, an experiment with the addition of 1% isopropyl alcohol (IPA) as a hydroxyl radical scavenger was performed to assess the role of OH radicals in the primary stages of ampicillin degradation. The influence of IPA addition on rate constants of AMP degradation is shown in Figure 5.

The lowest reduction in the rate constant due to the addition of IPA was observed for *R30* and amorphous TiO_2_ powders, suggesting that only approximately 30% of the degradation proceeds due to the involvement of mobile hydroxyl radicals. On the other hand, AMP degradation in the presence of *A100* and *A800* was strongly reduced (up to 90%) by IPA addition. A comparison of the results shows a good correlation between the particle size and the extent of adsorption for each photocatalytic material.

A kinetic study with *A5* and *A100* photocatalysts showed that with a similar extent of ampicillin degradation, the composition of the irradiated solutions differs after the addition of IPA. In the presence of IPA, the formation of products most likely due to oxidation by the mobile OH radical (intermediates designated I, V, VI and amoxicillin, AMX, Figure S1) is strongly suppressed and they are practically not observed in the chromatograms.

#### 3.3.3. DFT Study

Primary steps of ampicillin degradation were proposed and studied by quantum chemistry methods. In aqueous solutions, AMP is characterized by two dissociation constants, pK_a_ 2.5 and 7.1 [19]. As the zwitterionic form predominates during the experiments, it was used for calculations.

There are several reaction centers in AMP molecule suitable to interaction with OH radical or h^+^ (Figure 1). Four types of reactions can be distinguished: (i) H-abstraction by OH radical (reaction centers on C_2_, C_3a_, C_3b_, C_5_, C_6_, C_10_ atoms), (ii) addition of •OH on the aromatic ring (C_12_, C_13_, and C_14_), (iii) addition of •OH on carbonyl bond (C_7_, C_9_, C_15_), and (iv) the direct oxidation of AMP by photo formed valence band h^+^ on the photocatalyst surface. The primary steps with corresponding activation energies are summarized in Scheme S1.

**Figure 6:**
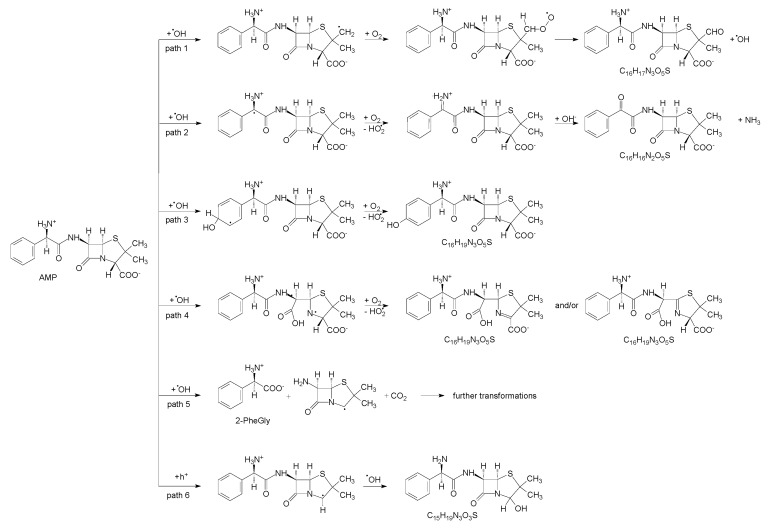
Mechanism of photocatalytic degradation of ampicillin including the identified primary products by UPLC/MS/MS.

By UHPLC/MS, a total of 5 products were found, which matched those predicted by DFT calculations. The analytes were identified according to their accurate mass (Figure S2). Most of them were eluted from a column at several different retention times, which may indicate the presence of isomeric forms. The presence of amoxicillin (*para*-hydroxylated AMP) and 2-phenylglycine were confirmed experimentally by co-injection with commercial standards. Figure 6 summarizes reaction pathways leading to the identified intermediates. Corresponding energetic profiles according to the reaction coordinate and optimized geometries of transition states and intermediates are shown in SI (Figures S3–S8).

Thus, ***path 1*** corresponds to the hydrogen abstraction by •OH from one of the methyl groups (atoms C_3a_ or C_3b_). The addition of O_2_ on C_3_ is followed by intramolecular hydrogen transfer, a strongly exothermic process resulting in the spontaneous elimination of •OH and stabilization of aldehydic intermediate C_16_H_17_N_3_O_5_S (M_w_ = 363.4).

***Path 2*** represents oxidative deamination of AMP to the product C_16_H_16_N_2_O_5_S (M_w_ = 348.4), initiated by H-abstraction by •OH from C_10_ atom. The following steps involve intermolecular transfer of hydrogen to O_2_ from the primary amino group that is oxidized to the imino group. The addition of the water molecule (hydroxide anion) to C_10_ and intramolecular hydrogen transfer to the nitrogen atom is terminated by spontaneous elimination of NH_3_. Oxidative deamination was observed by Klauson et al. [16] in the case of photocatalytic oxidation of AMX.

**Figure 7:**
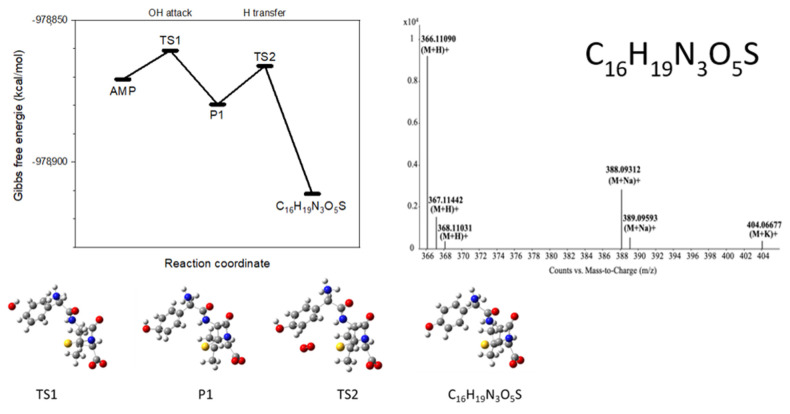
Characteristic reaction pathway in the degradation of ampicillin leading to the UPLC/MS/MS-identified Product.

In ***path 3***, the attack of the OH radical on the aromatic ring leads to the presence of oxygen to hydroxylated isomers, such as amoxicillin, i.e., *para*-hydroxylated AMP, C_16_H_19_N_3_O_5_S (M_w_ = 365.4). Intermediates with the same mass and elemental composition can be formed via ***path 4***, which is initiated by the addition of •OH to the β-lactam ring (atom C_7_) and its opening. The AMP-penicilloic acid radical can subsequently interact with O_2_ and after elimination of HO_2_•, one of the two depicted isomers is formed (Figure 7).

***Path 5*** corresponds to the •OH attack on the C_9_ carbonyl group, that leads to the cleavage of the AMP molecule into 2-phenylglycine (2-PheGly) and 6-aminopenicilanic acid (6-APA) radical which undergoes decarboxylation during its optimization.

Direct oxidation by h^+^ is presented in ***path 6***. Deprotonated AMP undergoes spontaneous decarboxylation, and formed radicals can straightforwardly react with •OH to C_15_H_19_N_3_O_3_S (M_w_ = 321.4). Oxidative decarboxylation and substitution by OH group was observed during photocatalytic degradation of other carboxylic acids [30,31].

## 4. Conclusions

The systematic research presented showed the correlations between the properties of both morphological (particle size, degree of agglomeration) and structure (crystallinity, allotropes) of TiO_2_ nanopowders, and their photocatalytic performance. The results showed there are linear correlation between crystallite size and two main performance characteristics, namely reaction rate constant and degree of mineralization. While the formed characteristic increased with crystallite size, the latter decreased. The two presented figures show that the reaction rate constant increases with increasing with crystallite size while the degree of mineralization decreases. These correlations are especially important as they are not predictable.

In our research, not only the photocatalysis itself but also accompanying processes, namely sorption on the photocatalytic surface in dark and photolysis, were addressed. The coupling of the experimental research and DFT calculations enabled to obtain a deep insight into the mechanism of the whole process, which is of major importance for the assessment of the effect of other components present simultaneously in the water (such as natural organic matter or inorganic salts). As the formation of potentially toxic organic products should be avoided, major attention was devoted to the determination of the photocatalyst properties, which are decisive for the achievement of complete mineralization. A further natural step in our research will be the inclusion of additional pharmaceuticals with different physico-chemical properties, which will enable not only to determine correlations between pharmaceuticals’ properties and the mechanism of their photocatalytic but also to achieve a more complex assessment of the potential of photocatalytic technology in the water purification [32].

## Figures and Tables

**Table 1:** Structure and texture properties of photocatalysts used for ampicillin degradation.

Photocatalyst Designation	Phase Content ^a^ (%)		Crystallite Size ^a^ (nm)		Particle Size ^b^ (nm)	Particle Size ^c^ (nm)
	Anatase	Rutile	Anatase	Rutile		
*A5*	100	0	13	-	13	13–30
*A15*	100	0	15	-	18	20–40
*A30*	100	0	16	-	31	20–40
*A100*	100	0	70	-	174	70–300
*A800*	100	0	58	-	156	50–300
*R30*	0	100	-	29	100	30–100
*R100*	0	100	-	nd	290	100–400
*R800*	0	100	-	nd	220	100–300
*A+R1*	55	45	32	62	92	30–150
*A+R2*	64	36	15	18	25	15–30
*A+R3*	31	69	16	18	36	15–30
*AM5*	100	0	5	-	6	5–10
*P25*	80	20			27	nd

^a^ from X-ray diffractograms, ^b^ from N_2_ sorption data, ^c^ from HR-TEM images, nd: not determined.

## Supplementary Materials

The following are available online at https://www.mdpi.com/article/10.3390/nano11081992/s1, Figure S1: HPLC analysis of irradiated suspensions of ampicillin (1 × 10^−4^ mol/L) with *A100* and *R30* (1 g/L), Figure S2: Mass spectra of intermediates detected by HPLC/MS after irradiation of AMP in suspension with anatase or rutile, Scheme S1: Primary OH radical attacks on ampicillin molecule. Values above and under arrows correspond to reaction energies of forward and reverse processes, resp., Figure S3: Changes of Gibbs relative energy along reaction coordinate and geometries of transition states and intermediates corresponding to the path 1 (Scheme in Figure 7), Figure S4: Changes of Gibbs relative energy along reaction coordinate and geometries of transition states and intermediates corresponding to the path 2 (Scheme in Figure 7), Figure S5: Changes of Gibbs relative energy along reaction coordinate and geometries of transition states and intermediates corresponding to the path 3 (Scheme in Figure 7), Figure S6: Changes of Gibbs relative energy along reaction coordinate and geometries of transition states and intermediates corresponding to the path 4 (Scheme in Figure 7), Figure S7: Changes of Gibbs relative energy along reaction coordinate and geometries of transition states and intermediates corresponding to the path 5 (Scheme in Figure 7), Figure S8: Changes of Gibbs relative energy along reaction coordinate and geometries of transition states and intermediates corresponding to the path 6 (Scheme in Figure 7).
