# Supplementary material for: Removal of Ampicillin by Heterogeneous Photocatalysis: Combined Experimental and DFT Study
Source: Nanomaterials (Basel). 2021 Aug 3;11(8):1992. doi: 10.3390/nano11081992 (PMC8399517; doi:10.3390/nano11081992)
Supplement: Supplementary file 1 [file nanomaterials-11-01992-s001.zip › nanomaterials-1327541-supplementary.pdf]

nanomaterials

Supplementary Materials

Removal of Ampicillin by Heterogeneous Photocatalysis: Combined Experimental and DFT Study

Lenka Belhacova [1,*], Hana Bibova [1], Tereza Marikova [2,3], Martin Kuchar [3], Radek Zouzelka [2] and Jiri Rathousky [2,*]

[1] Department of Electrochemical Materials, J. Heyrovsky Institute of Physical Chemistry of the CAS, Dolejskova 3, 18223 Prague, Czech Republic; hana.bibova@jh-inst.cas.cz
[2] Center for Innovations in the field of Nanomaterials and Nanotechnologies, J. Heyrovsky Institute of Physical Chemistry of the CAS, Dolejskova 3, 18223 Prague, Czech Republic; tereza.marikova@jh-inst.cas.cz (T.M.); radek.zouzelka@jh-inst.cas.cz (R.Z.)
[3] Forensic Laboratory of Biologically Active Substances, Department of Chemistry of Natural Compounds, University of Chemistry and Technology Prague, Technicka 3, 16628 Prague, Czech Republic; kuchara@vscht.cz
* Correspondence: lenka.belhacova@jh-inst.cas.cz (L.B.); jiri.rathousky@jh-inst.cas.cz (J.R.)

1. Comparative Kinetic Study with *A100* and *R30* Powders

A comparative kinetic study was performed with *A100* and *R30* powders. Seven major peaks corresponding to the primary intermediates were identified (Figure S1), the detailed identification of which will be the subject of the following publication. Their concentration was estimated for comparative reasons with an assumption that as primary intermediates, they have similar structure and therefore spectral properties (extinction coefficient at the detection wavelength) as ampicillin. By nonlinear regression analysis, relative (and mutually comparable) values of the rate constants of formation and degradation of each intermediate were calculated, Table S1.

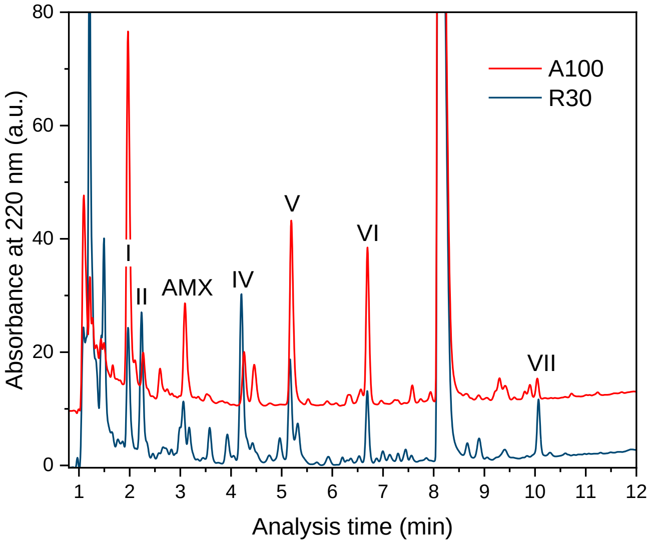

Figure S1. HPLC analysis of irradiated suspensions of ampicillin (1x10^{-4} mol/L) with *A100* and *R30* (1g/L).

Table S1. Ratio of a given intermediate (first column) formation rate constant to the AMP degradation rate constant for *R30* and *A100* photocatalysts.

	**Rutile**	**Anatase**
I	7.8	15.3
II	4.7	1.7
AMX	3.9	6.3
IV	7.9	6.0
V	8.1	11.4
VI	3.8	7.9
VII	19.1	1.1

2. Mass Spectra of Identified Products

The intermediate with Mw=381.4 and formula $C_{16}H_{19}N_3O_5S$ corresponds to the secondary product resulting from two attacks of OH radical. Its formation can be described as follows:

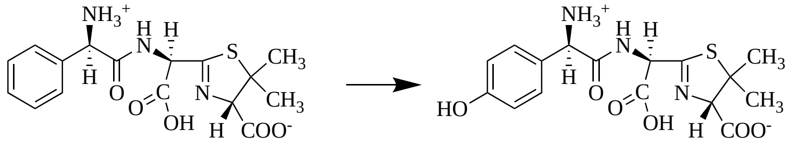

($C_{16}H_{19}N_3O_5S$) ($C_{16}H_{19}N_3O_6S$)

Figure S2. Mass spectra of intermediates detected by HPLC/MS after irradiation of AMP in suspension with anatase or rutile.

3. Mechanism of Photocatalytic Degradation of Ampicillin - Theoretical Study of Primary Reaction Steps

Detailed computational study revealed several reaction centers on ampicillin molecule, susceptible to interaction with hydroxyl radical. Primary OH radical attacks are shown in Scheme S1 with reaction energies of forward and reverse processes.

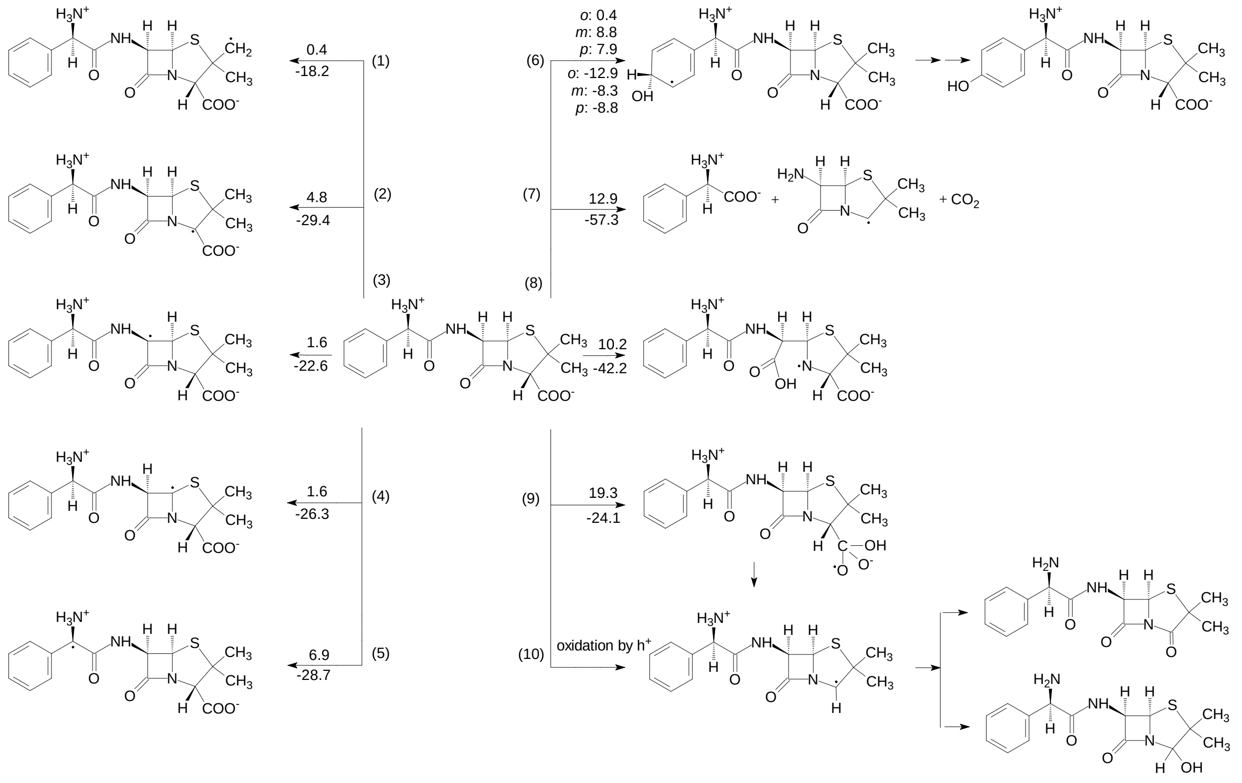

Scheme S1. Primary OH radical attacks on ampicillin molecule. Values above and under arrows correspond to reaction energies of forward and reverse processes, resp.

The OH radical attacks run fast and irreversibly due to low activation energies in the forward direction and the relatively high activation energies of the corresponding reverse reactions. Deprotonated form of AMP adsorbed on the photocatalyst surface interacts with a photo formed valence band h^+ under formation of a radical that undergoes spontaneous decarboxylation. In the presence of triplet oxygen, subsequent processes (often spontaneous) follow, terminated by a release of OH or HO_2 radicals to form stable primary intermediates.

The following reaction pathways leading to the products identified by HPLC/MS are discussed in detail:

Path 1 (Figure S3) corresponds to the hydrogen abstraction by •OH from one of the methyl groups (atoms C_{3a} or C_{3b}) under the release of H_2O molecule (TS1 and formed radical intermediate P1). Addition of O_2 on C_3 (P2, no transition state found) is followed by intramolecular hydrogen transfer, a strongly exothermic process (TS2) resulting in the spontaneous elimination of •OH and stabilization of aldehydic intermediate $C_{16}H_{17}N_3O_5S$ (M_w = 363.4).

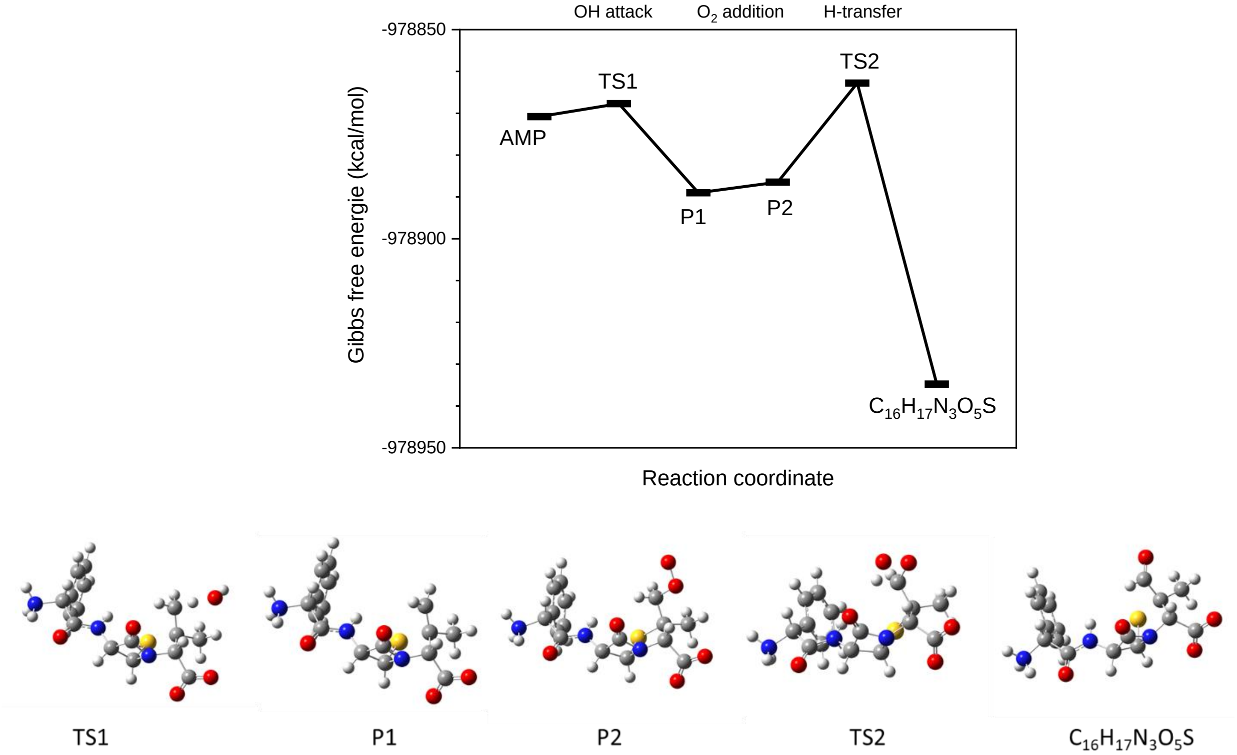

Figure S3. Changes of Gibbs relative energy along reaction coordinate and geometries of transition states and intermediates corresponding to the path 1 (Scheme in Figure 7).

Path 2 (Figure S4) represents oxidative deamination of AMP to the product $C_{16}H_{16}N_2O_5S$ (M_w = 348.4), initiated by H-abstraction from C_{10} atom (TS1, P1). In the next step, addition of O_2 on this reaction center proceeds (TS2, P2), followed by intermolecular transfer of H atom from the primary amino group to O_2, resulting in oxidation to the imino group and release of HO_2• (TS3, P3). After addition of water molecule (hydroxide anion) to C_{10} (P4), intramolecular hydrogen transfer to nitrogen atom is terminated by spontaneous elimination of NH_3. Involvement of the molecule of water (water catalyzed reaction, TS4) reduces activation energy from 28.1 kJ/mol to energetically much feasible value of 12.4 kJ/mol.

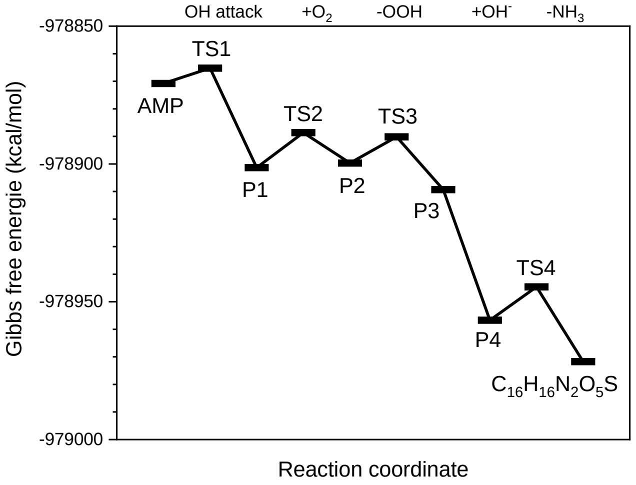

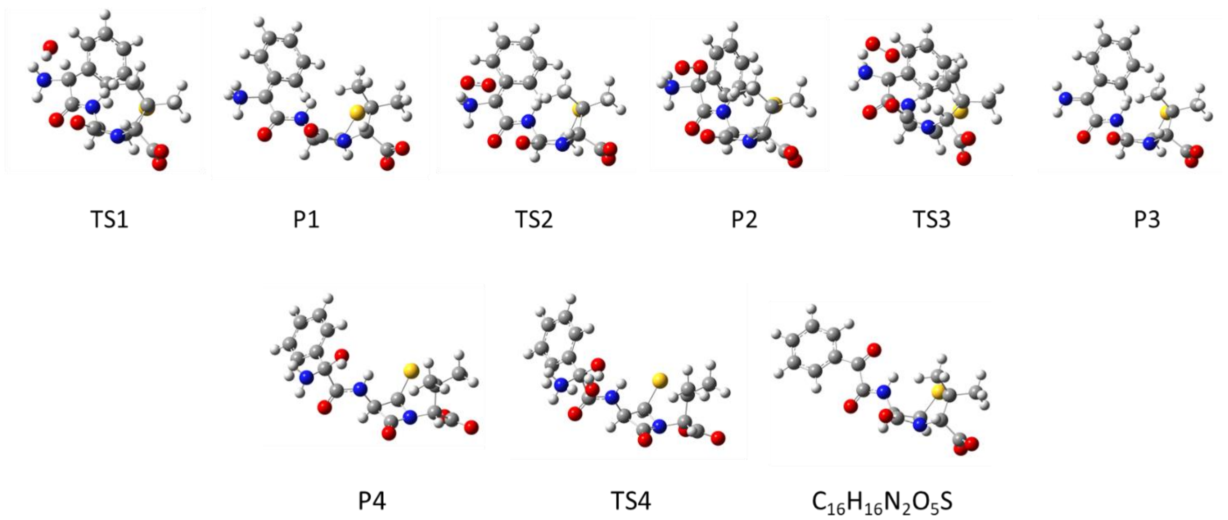

Figure S4. Changes of Gibbs relative energy along reaction coordinate and geometries of transition states and intermediates corresponding to the path 2 (Scheme in Figure 7).

Path 3 (Figure S5) shows the formation of amoxicillin. The process is initiated by the attack of •OH on the aromatic part of AMP (TS1). Formed OH-adduct (P1) interacts with O_2 *via* hydrogen transfer (TS2) and restores its aromatic character under release of HO_2•, leaving a hydroxylated isomer of AMP. If the OH attack is directed to *para* position, amoxicillin, i.e., *para*-hydroxylated AMP, $C_{16}H_{19}N_3O_5S$ (M_w = 365.4), is formed.

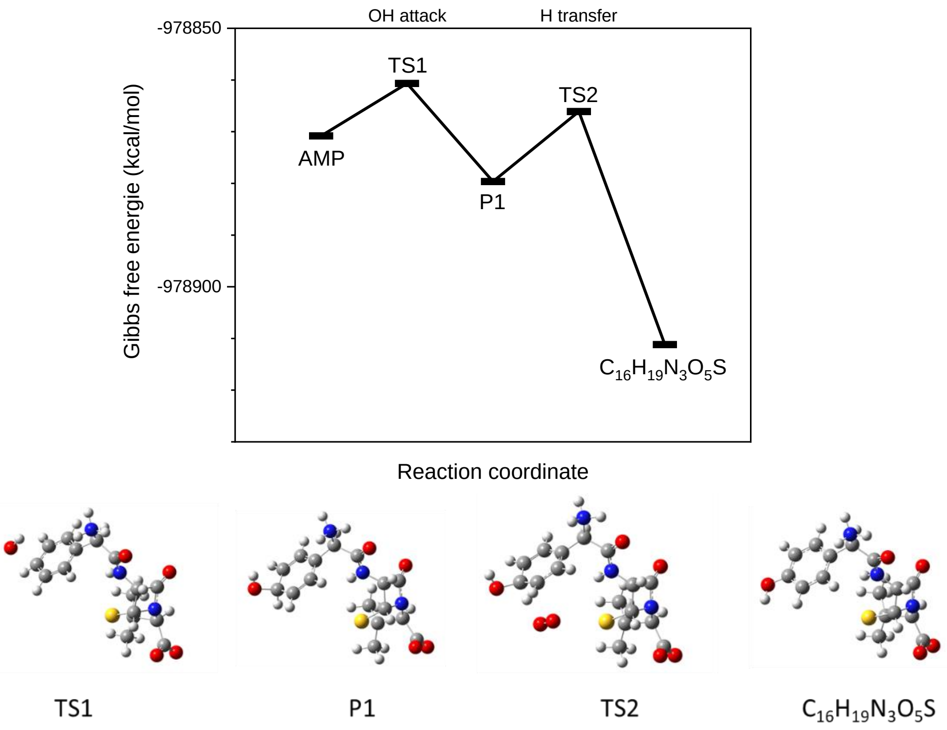

Figure S5. Changes of Gibbs relative energy along reaction coordinate and geometries of transition states and intermediates corresponding to the path 3 (Scheme in Figure 7).

Path 4 (Figure S6) leads to another product with the same mass and elemental composition as in the previous path, $C_{16}H_{19}N_3O_5S$ (M_w = 365.4). The transformation is initiated by OH attack on carbonyl group (atom C_7) of β-lactam ring (TS1), resulting in its opening (P1). The AMP-penicilloic acid radical (P1) can subsequently interact with O_2 (TS2) and then undergo H-transfer from an adjacent carbon atom (C_2 or C_5; TS3 corresponds to C_2 alternative) to P3 radical intermediate. After an intramolecular rearrangement (TS4, P4) elimination of HO_2• (TS5), one of the two isomers is formed.

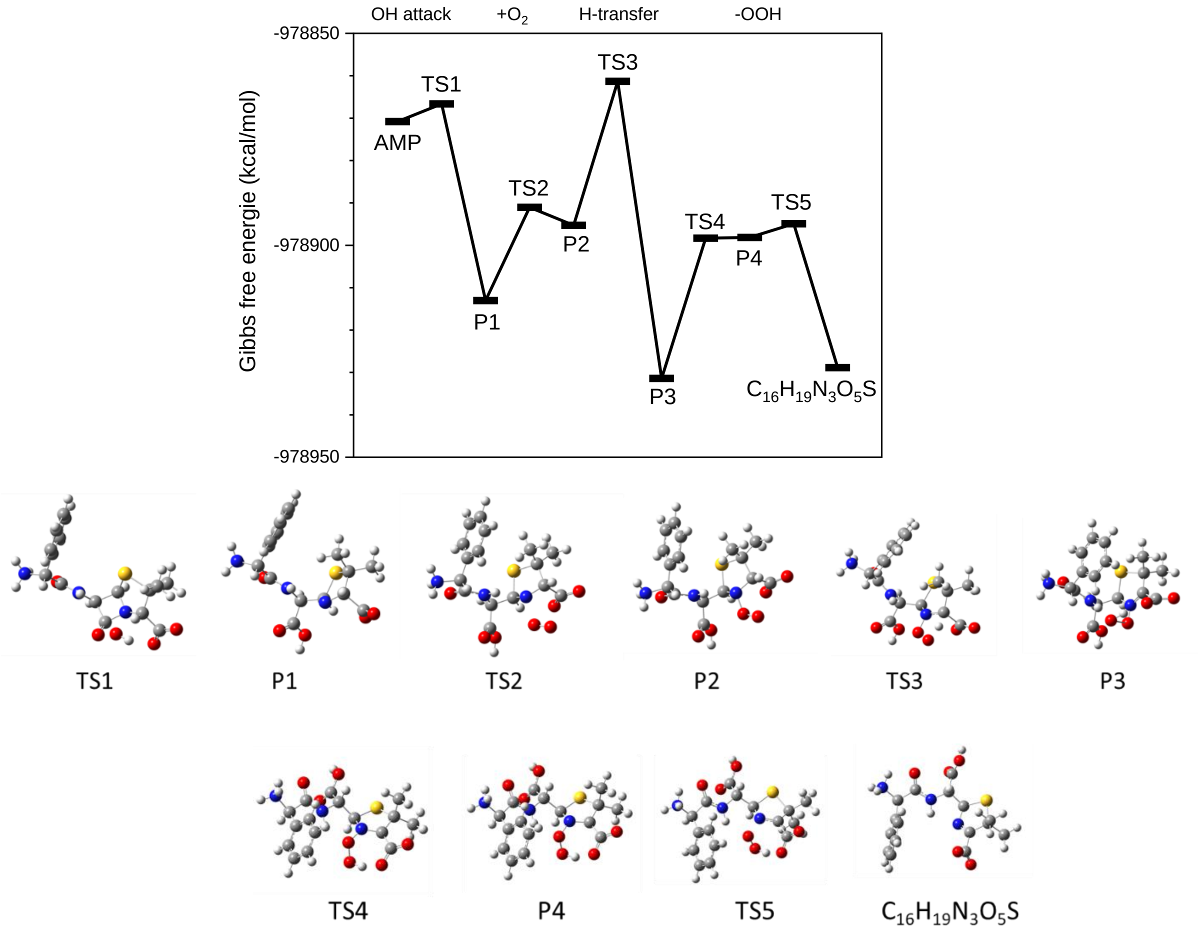

Figure S6. Changes of Gibbs relative energy along reaction coordinate and geometries of transition states and intermediates corresponding to the path 4 (Scheme in Figure 7).

Path 5 (Figure S7) corresponds to the •OH attack on the C_9 carbonyl group leading to the cleavage of the AMP molecule to the precursors of its synthesis, 2-phenylglycine (2-PheGly) and 6-aminopenicilanic acid (6-APA) radical. The calculations show that the spin density of the 6-APA radical is localized on N_8 atom, which will be most likely a center of subsequent reactions.

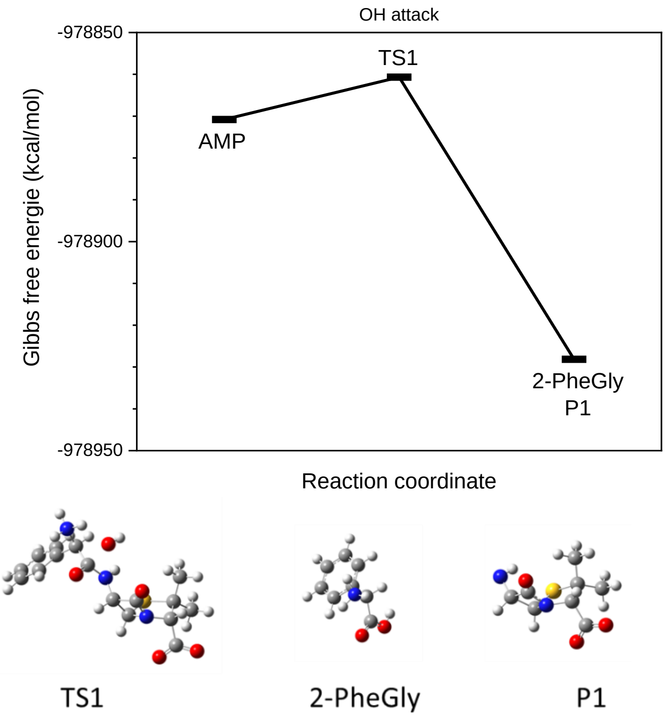

Figure S7. Changes of Gibbs relative energy along reaction coordinate and geometries of transition states and intermediates corresponding to the path 5 (Scheme in Figure 7).

Path 6 (Figure S8) corresponds to the direct oxidation of adsorbed AMP anion by a positive hole (h+) localized on the surface resulting in spontaneous decarboxylation (P1). The radical can further spontaneously react with present hydroxyl radical to form $C_{15}H_{19}N_3O_3S$ (M_w = 321.4). Oxidative decarboxylation and substitution by OH group was observed during photocatalytic degradation of other carboxylic acid (Ranjit et al., 2001, Romeiro et al. 2018).

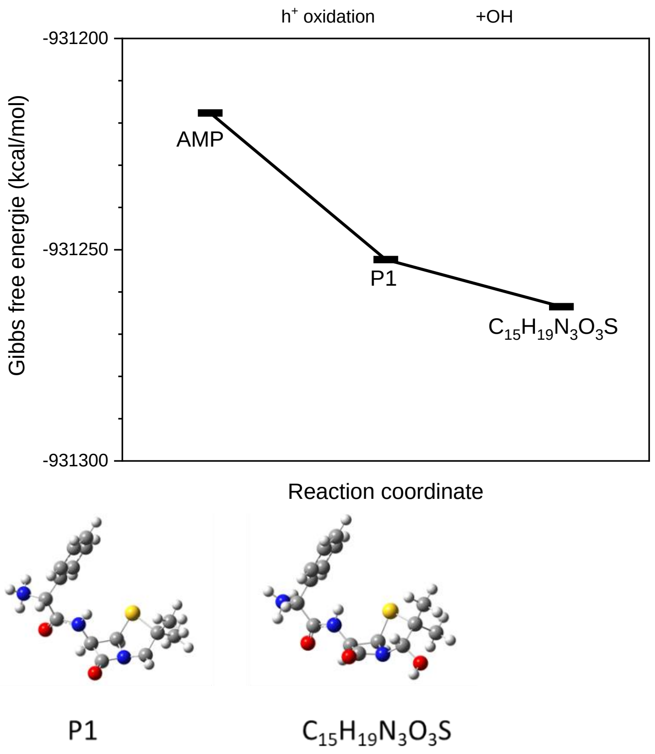

Figure S8. Changes of Gibbs relative energy along reaction coordinate and geometries of transition states and intermediates corresponding to the path 6 (Scheme in Figure 7).

4. Correlation between Photocatalytic Performance and the Crystallite Size of Anatase Nanopowder

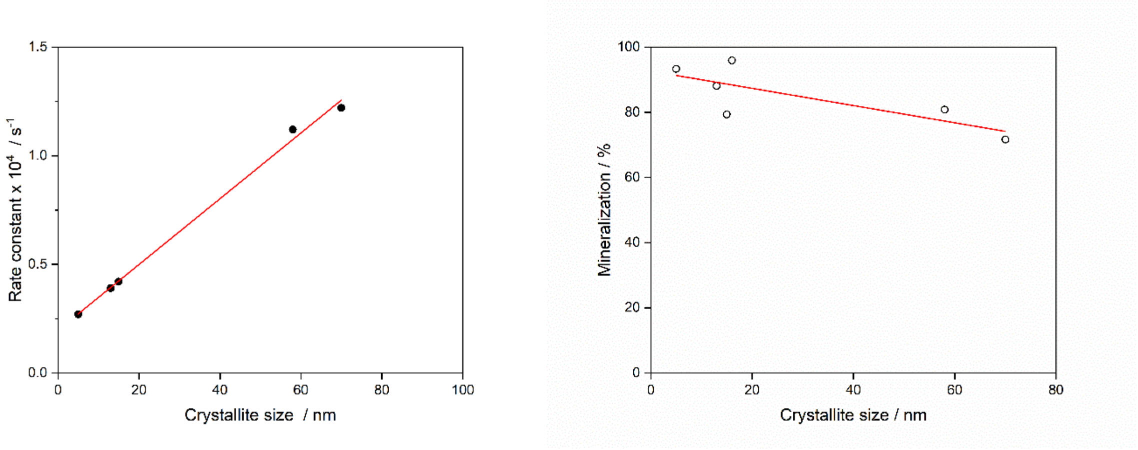

Figure S9. Correlation between reaction rate constant (left) and degree of mineralization (right) and anatase crystallite size.

From our experiments clear cut correlations follow. The most important are the correlation between crystallite size vs. reaction rate constant and degree of mineralization vs. reaction rate constant calculated for anatase samples. These two parameters have fundamental importance for the assessment of the photocatalytic activity of anatase nanopowders. The reason for the selection of anatase nanopowders was their high activity and sufficiently extensive series of tested samples. Furthermore, the crystallite size determined from X-ray diffraction is the property which has the most important effect on the photocatalytic performance.

The two presented figures show that the reaction rate constant increases with increasing crystallite size, while the degree of mineralization decreases.
